# Ablation of the integrin CD11b mac-1 limits deleterious responses to traumatic spinal cord injury and improves functional recovery in mice

**DOI:** 10.21203/rs.3.rs-4196316/v1

**Published:** 2024-04-04

**Authors:** Yun Li, Rodney M. Ritzel, Junyun He, Simon Liu, Li Zhang, Junfang Wu

**Affiliations:** University of Maryland School of Medicine; University of Maryland School of Medicine; University of Maryland School of Medicine; University of Maryland School of Medicine; University of Maryland School of Medicine; University of Maryland School of Medicine

**Keywords:** Spinal cord injury, Mac-1/CD11b, microglia/macrophage, neuroinflammation

## Abstract

**Background:**

Spinal cord injury (SCI) causes long-term sensorimotor deficits and posttraumatic neuropathic pain, with no effective treatment. In part, this reflects an incomplete understanding of the complex secondary pathobiological mechanisms involved. SCI triggers microglial/macrophage activation with distinct pro-inflammatory or inflammation-resolving phenotypes, which potentiate tissue damage or facilitate functional repair, respectively. The major integrin Mac-1 (CD11b/CD18, αMβ2 or CR3), a heterodimer consisting of αM (CD11b) and β2 (CD18) chains, is generally regarded as a pro-inflammatory receptor in neurotrauma. Multiple immune cells of the myeloid lineage express CD11b, including microglia, macrophages, and neutrophils. In the present study, we examined the effects of CD11b gene ablation on posttraumatic neuroinflammation and functional outcomes after SCI.

**Methods:**

Young adult age-matched female CD11b knockout (KO) mice and their wildtype (WT) littermates were subjected to moderate thoracic spinal cord contusion. Neuroinflammation in the injured spinal cord was assessed with qPCR, flow cytometry, NanoString, and RNAseq. Neurological function was evaluated with the Basso Mouse Scale (BMS), gait analysis, thermal hyperesthesia, and mechanical allodynia. Lesion volume was evaluated by GFAP-DAB immunohistochemistry, followed by analysis with unbiased stereology.

**Results:**

qPCR analysis showed a rapid and persistent upregulation of CD11b mRNA starting from 1d after injury, which persisted up to 28 days. At 1d post-injury, increased expression levels of genes that regulate inflammation-resolving processes were observed in CD11b KO mice. Flow cytometry analysis of CD45^int^Ly6C^−^CX3CR1^+^ microglia, CD45^hi^Ly6C^+^Ly6G^−^ monocytes, and CD45^hi^Ly6C^+^Ly6G^+^ neutrophils revealed significantly reduced cell counts as well as reactive oxygen production in CD11b KO mice at d3 post-injury. Further examination of the injured spinal cord with NanoString Mouse Neuroinflammation Panel and RNAseq showed upregulated expression of pro-inflammatory genes, but downregulated expression of the reactive oxygen species pathway. Importantly, CD11b KO mice exhibited significantly improved locomotor function, reduced cutaneous mechanical/thermal hypersensitivity, and limited tissue damage at 8 weeks post-injury.

**Conclusion:**

Collectively, our data suggest an important role for CD11b in regulating tissue inflammation and functional outcome following SCI. Thus, the integrin CD11b represents a potential target that may lead to novel therapeutic strategies for SCI.

## Introduction

Spinal cord injury (SCI) is a devastating event that leads to long-term disability and even life-threatening consequences. After the initial mechanical damage of spinal cord, secondary injury processes produce a cascade of biochemical changes within the lesion site and surrounding tissue. These delayed and often long-lasting processes include activation of microglia and astrocytes, infiltration of macrophages and other peripheral immune cells, overproduction of reactive oxygen species (ROS), and neurotoxicity (1–3), leading to extended tissue loss and neurological dysfunction. Following activation, microglia/macrophages present distinct disease-associated phenotypes shortly after injury: pro-inflammatory and inflammation-resolving (4). The former can exacerbate tissue damage, while the latter can potentiate functional recovery. This process is driven by the complex interaction between the central nervous system (CNS)’s resident microglia and infiltrated immune cells. However, the underlying mechanisms for phenotype determination are still not fully understood.

Many studies have demonstrated that integrins, a large family of transmembrane proteins, regulate the interaction between immune cells and their extracellular environment, thus play a major role in driving the polarization of macrophages and microglia (5–8). Examples include microglia/macrophage beta2 integrins binding to vascular adhesion molecules in the vascular lumen and promoting migration toward the injury site (9). Using monoclonal antibodies, studies showed that blocking integrin αDβ2 (CD11d/CD18) activity reduced intraspinal inflammation, improved neurological functions, and was able to attenuate systemic inflammatory syndrome after SCI (9–12). Moreover, β1-integrin has been reported to regulate astrogliosis and glial scar formation following SCI (13), suggesting that immune modulation through the integrin family is a potential route for future strategies. More recently, studies have shown that inhibition of integrin beta1 and its downstream signaling pathway can improve blood spinal cord barrier repair and promote neurological recovery after trauma (14, 15).

The major integrin CD11b/CD18 (also known as Mac-1, αMβ2 and CR3) is a heterodimer of CD11b (αM) and CD18 (β2) subunits (16, 17). The CD11b/CD18 integrin is highly expressed in all myeloid lineage immune cells, including microglia, macrophages, and neutrophils. As early as 1999, a study has reported on the expression levels of intercellular adhesion molecule 1 (ICAM-1) and CD11b in the acute stages of CNS after mechanical compression, showing significant upregulation in both the lesion center and perifocal zones within the first week of injury (18). More recently, studies using mouse models of optic nerve injury, hypertensive cardiac remodeling, liver fibrosis, pneumococcal pneumonia, and others have shown that CD11b knockout (KO) can promote debris clearance, attenuate neural degradation, and reduce macrophage infiltration (19–23). The extensive reporting on the role that CD11b/CD18 integrin plays in regulating myeloid cells has led us to explore the possibility of using CD11b as a therapeutic target in SCI. Prior work has shown that CD11b ablation can have neuroprotective effects in CNS diseases (24–27). However, CD11b also mediated anti-inflammatory activity following activated protein C (APC) treatment of sepsis (28–31). Thus far, the specific role of CD11b in SCI pathophysiology has yet to be fully addressed.

Here we utilize a contusion mouse SCI model to examine cellular and molecular changes as well as behavior and histopathology in the absence and presence of CD11b gene. We demonstrate that SCI-induced acute inflammatory response is increased, whereas production of free oxygen species in microglia/macrophages/neutrophils is reduced in the CD11b KO mice, as measured by qPCR, flow cytometry, NanoString, and bulk RNAseq analysis. Importantly, we report that functional deficits in locomotion and hypersensitivity to mechanical and thermal stimuli are significantly limited in CD11b KO mice and were associated with reduced tissue damage. Together, our studies demonstrate that CD11b regulates the status of beneficial neuroinflammation in the early-phase response to SCI, potentially contributing to SCI pathophysiology.

## Materials and Methods

### Animals and mouse spinal cord contusion model

All surgical and experimental procedures in this study were performed under protocols approved by the Institutional Animal Care and Use Committee (IACUC) at the University of Maryland School of Medicine. To reduce the risk of bladder infection (32, 33), we only used female mice in this study. Young adult (10–12 weeks) female CD11b KO mice were obtained from Dr. Li Zhang’s lab and maintained in the UMB animal facility. Following the administration of isoflurane anesthesia, a laminectomy was carried out. The spinal column was then secured by attaching metal clamps to the lateral processes at the T9 and T11 levels. The mice were subjected to a midline contusion injury of the spinal cord at the T10 level using the Infinite Horizon Spinal Cord Impactor (Precision Systems and Instrumentation) with a force of 60 kilodyne, which is classified as moderate injury (34, 35). For the first 7–14 days after SCI, manual bladder expression was carried out at least three times per day until reflex bladder emptying was re-established. For mice used as control animals, sham surgery was performed after anesthesia, consisting of only a laminectomy without contusion. Individuals who performed functional assessment and data analysis were blinded to group designations and genotypes throughout all stages of the experiment.

### Flow cytometry

Following euthanization, mice were perfused with 40 mL of ice-cold PBS, and a segment (5 mm length) of spinal cord tissue surrounding the lesion area was isolated, weighed, and placed in complete Roswell Park Memorial Institute (RPMI) 1640 (Cat# 22400105, Invitrogen) medium with 10% fetal bovine serum (FBS). The tissue samples were mechanically and enzymatically digested in collagenase/dispase (Cat# 10269638001, 1 mg/ml; Roche Diagnostics), papain (Cat# LS003119, 5 U/ml; Worthington Biochemical), 0.5 M EDTA (Cat# 15575020, 1:1000; Invitrogen), and DNAse I (Cat# 10104159001, 10 mg/ml; Roche Diagnostics) for 1h at 37°C on a shaking incubator (200 rpm). The cell suspension was washed twice with RPMI and filtered through a 70-μm cell strainer before a final volume of 3 ml was set by adding RPMI and kept on ice. The spinal cord cells were then transferred to FACS tubes and washed with FACS buffer, followed by incubation with Fc Block (Cat# 101320, Clone: 93; Biolegend) for 10 min on ice. The spinal cord cells were stained with the following surface antigens: [CD45-eF450 (Cat# 48-0451-82, Clone: 30-F11; eBioscience), CD11b-APC/Fire^™^750 (Cat# 101262, Clone: M1/70; Biolegend), Ly6C-APC (Cat# 128016, Clone: HK1.4; Biolegend), Ly6G-AF700 (Cat# 128024, Clone: 1A8; Biolegend), Cx3CR1-PE (Cat# 149006, Clone: SA011F11, Biolegend)] and Zombie Aqua fixable viability dye (Cat# 423102, Biolegend). The spinal cord cells were then washed in FACS buffer, fixed in 2% paraformaldehyde (PFA) for 8 min, and washed once more prior to adding 500 μl FACS buffer. For measurement of ROS production, we used H_2_DCFDA (DCF, Cat# C6827, 5 μm; Thermo Fisher Scientific) as previously described (36–38). In brief, the DCF dye was added to RPMI media according to the manufacturer’s instructions, vortexed and incubated for 30 min in a 37°C water bath. All data was acquired on a BD LSRFortessa cytometer FACSDiva 6.0 (BD Biosciences) and analyzed using FlowJo (Treestar Inc.), with a minimum number of 5 million events collected for each sample. Countbright^™^ Absolute Counting Beads (Cat# C36950; Invitrogen) were used to estimate cell counts per the manufacturer’s instructions. Cell count data was expressed as total counts/mg spinal cord tissue weight. Leukocytes were first gated using a splenocyte reference (SSC-A vs. FSC-A), followed by singlets (FSC-H vs. FSC-W) and live cell gating based upon exclusion of Zombie Aqua (SSC-A vs. Zombie Aqua-Bv501). Resident microglia were identified as the CD45^int^Ly6C^−^CX3CR1^+^ population, whereas peripheral myeloid cells were identified as the CD45^hi^Ly6C^+^CX3CR1^+^ population. Within the myeloid subset, further separation was achieved by identifying monocytes as Ly6C^hi^Ly6G^−^ and neutrophils as Ly6C^+^Ly6G^+^. Cell type-matched fluorescence minus one (FMO) controls were used to determine the positivity of each antibody and the DCF dye (39).

### Total RNA extraction and real-time PCR (qPCR)

RNA samples were obtained after sample processing with the RNeasy mini kit (Cat# 74104, Qiagen) from 5 mm of spinal cord tissue at various time-points post-injury. Complementary DNA (cDNA) was synthesized by a Verso cDNA RT kit (Cat# AB1453B, Thermo Scientific) per the manufacturer’s protocol. Real-time PCR for target mRNAs was performed with TaqMan gene expression assays for Itgam (Mm00434455_m1), CD83 (Mm01350412_m1), P2yr12 (Mm01950543_s1), Tmem119 (Mm00525305_m1), Trem2 (Mm04209424_g1), Gfap (Mm01253033_m1), Gdf15 (Mm00442228_m1), Opalin (Mm00463365_m1), Fcrls (Mm01219428_m1), Cxcl10 (Mm00445235_m1), Fkbp5 (Mm00487406_m1), H2-T23 (Mm00439246_g1), Csf1r (Mm01266652_m1), Pllp (Mm00452740_m1), Ennp6 (Mm00624107_m1), Plekhm1 (Mm00805590_m1).

### Nanostring Analysis

RNA samples were acquired from 5 mm of spinal cord tissue one day after SCI. We used the NanoString nCounter^®^ system to analyze total RNA (20ng/ul) and obtain the transcript counts for 757 genes and 13 housekeeping genes from the Mouse Neuroinflammation panel (NanoString Technologies, Seattle, WA). The gene transcript counts were normalized before further analysis, and paired differential expression analysis was carried out using NanoString’s nSolver software Version 4.0. The statistical analysis of NanoString data was conducted using R language (R version 4.2.2) in RStudio version 2023.6.0, build 421. Partial least square discriminant analysis (PLS-DA) was performed with the Mixomics package (40), while pathway enrichment analysis was performed with the online Enrichr toolkit from Maayan Lab (41, 42). All plots were drawn with the ggplot2 package (43), with volcano plots and heatmaps being rendered by the EnhancedVolcano and ComplexHeatmap (44) packages, respectively.

### Bulk RNA sequencing and transcriptomic analysis

Total RNA was extracted using RNeasy mini kit (Qiagen) and sent to Novogen (Sacramento, CA) for RNA quality test and RNA-seq. The RNA quality was initially analyzed using Nanodrop and Agarose Gel Electrophoresis, and the quality of cDNA library is analyzed using an Agilent 2100 Bioanalyzer. RNA-seq transcriptomic analysis of all libraries was performed on Illumina, with data being converted into FastQ format for downstream analysis. The RNAseq results were validated for selected genes using reverse-transcription real-time quantitative PCR (RT-qPCR). Bioinformatics analyses were performed by Novogen in RStudio version 1.2 with R v3.6.1. The FastQC v0.11.8 package (45) was used for data quality assessment and [ribosomal RNA were filtered with SortMeRNA (46). For quantification of transcript-level abundance, the quasi-mapping-based mode was used in Salmon v1.0.0 (47), with mapping to GRCm38 (mm10) mouse reference genome. Transcript-level abundance was aggregated gen-level abundance with tximport (48), while the package DESeq2 (49) was used for differential expression analysis. Volcano plots and heatmaps were rendered with the EnhancedVolcano and ComplexHeatmap (44) packages in Rstudio. Pathway enrichment analysis was performed with the online Enrichr toolkit from Maayan Lab (41, 42).

### Tissue processing and histological analysis

Mouse spinal cord was dissected out following intracardial perfusion with ice-cold normal saline and 4% paraformaldehyde. The segments containing the lesion area, or an equal length of spinal cord for sham mice, were embedded in Optimal Cutting Temperature (O.C.T) compound, and cut into 20-μm-thick serial sections. In the assessment of lesion volume, sections spaced 1 mm apart within a segment of 5 mm on each side from rostral to caudal of the injury epicenter were stained with GFAP (1:1000; Cat# Z0334, Dako) and DAB (Cat# PK-6100, Vector Labs) as the chromogen. The Cavalieri method was used with Stereo Investigator Software (MBF Biosciences) for quantification as described in previous publications (38, 50).

### Neurological behavioral tests

Basso mouse scale (BMS) for locomotion: To evaluate locomotor function using BMS (51), mice were placed on a level, enclosed surface of 100 cm in diameter. Two trained researchers, unaware of the genotype of each mouse, observed them for at least 4 minutes. The animals were evaluated on a scale ranging from 0 to 9, where 0 indicated total paralysis of the hind limbs and 9 indicated normal locomotor activity. The grading criteria are derived from the evaluation of hind limb joint mobility, weight distribution, plantar stepping, and coordination. The mice underwent BMS score testing on day 1, day 3, and at weekly intervals for up to 6 weeks after SCI.

Catwalk XT automated gait analysis: The CatwalkXT automated system (Noldus; RRID:SCR_004074) was used for gait analysis as described previously (35, 37, 52). Each mouse underwent a single testing session, six weeks after SCI, to encourage exploration of the CatWalk while maintaining situational novelty. The CatWalk apparatus itself features a glass walkway emitting green LED light, which is refracted on the areas where the paws touch. A computer-operated high speed color camera captures the areas of contact and records the data in the CatWalk XT software. Data acquisition was performed in a darkened room by a single researcher blinded to the mice genotype and handling each subject. Animals were first placed in the open end of the CatWalk and allowed to walk across the walkway to the darkened escape enclosure. A minimum of three valid runs, or complete walkway crossings, were obtained for each subject. Trials in which the animal stopped partway across or turned around during a run were excluded from analysis.

Hot plate test: To test SCI-induced cutaneous hypersensitivity of the hind paws, mice were placed on the contact probe of computerized thermal stimulator on an Incremental Hot/Cold Plate Analgesia Meter (PE34, IITC Life Science, Woodland Hills, CA). The temperature was increased from 30 to 50°C with the incremental rate at 10°C per minute. When the tested mouse licked either one of its hind paws, the test was stopped, and the threshold temperature was recorded. The test was conducted twice with the interval of 3 h and the average stop temperature and latency time was recorded.

Von Frey test: The von Frey filament method was used to detect hind-paws withdrawal from a mechanical stimulus according to the “up-down” Von Frey method outlined by Caplan et al (53) and simplified by Bonin et al (54). At the start of the experiment, each mouse was individually placed in Plexiglass cubicles on a wire mesh platform and allowed to acclimate for 30 minutes. The von Frey filaments (MyNeuroLab, St. Louis, MO), with incremental stiffness ranging from 0.04 g to 2.0 g, were applied to the plantar surface of each hind paw. The experiment tests the response of the hind paw, with the first stimulus being set at a force estimated to be close to the 50% withdrawal threshold (0.4g). A positive response is defined as a brisk paw withdrawal (at least 3 times out of 5 applications) in response to the filament, while no paw withdrawal was considered a negative response. If there is no response, the next filament with a higher force is tested; if there is a response, the next lower force filament is tested. This continues until 20 readings are obtained for each paw, and the sequence of outcomes (− for no response or + for response) is recorded. The statistical formula published by Dixon et al was used to determine the mechanical force required to elicit a paw withdrawal response in 50% of animals (55). The average 50% pain threshold across three trials (one trial per day) was used to obtain an accurate estimate of mechanical force threshold.

### Statistical analysis

Quantitative data are all plotted as mean ± standard error of mean, and individual data point is shown. The animal numbers required for each experiment were derived from variability estimated from published data and power calculations based on effect sizes defined by Cohen (56). All statistical analyses were conducted with GraphPad Prism (Version 9.00) for Windows (GraphPad Software; RRID: SCR_002798). Two-way ANOVA with repeated measures was used to analyze BMS scores, followed by Sidak’s multiple comparisons post hoc test. One-way or two-way ANOVA was used for multiple comparisons between groups, followed by Tukey’s multiple comparisons post hoc test parametric data (normality and equal variance passed). For lesion volume, stereological data was analyzed using Student’s t-test. Statistical analysis in each assay was detailed in figure legends. A p-value of ≤ 0.05 was considered statistically significant.

## Results

### Ablation of CD11b gene limits acute proinflammatory response to SCI

**Figure 1:**
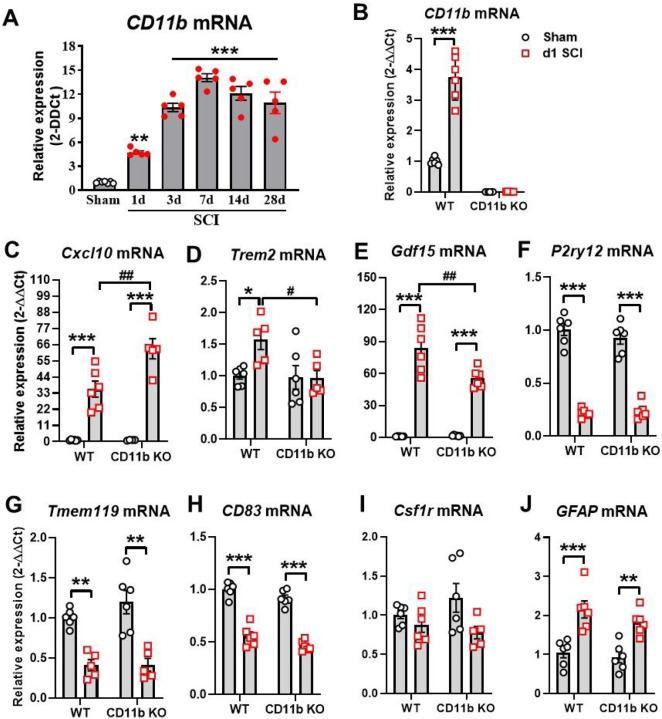
CD11b mediates acute inflammatory response to SCI. **(A)** qPCR analysis was performed to examine the mRNA expression of CD11b in the injured spinal cord of C57BL/6 WT female mice. n=7 (Sham) and 5 (SCI). **p<0.01, ***p<0.001 vs. Sham group. One-way ANOVA following Dunnett’s multiple comparisons test. (B-J) qPCR analysis was used to examine inflammatory responses in the injured spinal cord of young adult CD11b KO and WT female mice at 1d SCI. *p<0.05, **p<0.01, ***p<0.001 vs. Sham/WT. #p<0.05, ##p<0.01 vs. SCI/WT. n=5–6/group. Two-way ANOVA followed by Tukey’s post hoc test.

As a first step in exploring the role that CD11b plays in acute neuroinflammation, we used qPCR analysis to examine the changes to its expression level at multiple timepoints after SCI. We observed a significant increase of CD11b mRNA expression starting at 1 d post-injury, reaching its peak at 7 d and remained persistently high for up to 28 d (Fig. 1A). Next, we examined the effects of CD11b ablation on microglia and monocyte markers, along with genes that reflect their phenotypic functions. At 1d after SCI, qPCR analysis showed near-zero levels of CD11b (*Itgam*) mRNA expression in both Sham and SCI of CD11b KO mice, as expected (Fig. 1B). Furthermore, the gene *Cxcl10*, a chemokine well-known for inducing microglia migration and initiating microglial activation (57), showed significant increases in the injured spinal cord of both genotype groups, with CD11b KO mice showing even higher levels (Fig. 1C). In contrast, the gene *Trem2*, which is expressed in microglia and other myeloid cells of the CNS, showed a marked increase in SCI/WT mice, but no injury-induced changes were observed in CD11b KO mice (Fig. 1D). As a member of the transforming growth factor beta superfamily and known as macrophage inhibitory cytokine-1 (MIC-1), the expression levels of *Gdf15* were very low in both sham groups but showed significant increases after injury. Pairwise comparison of SCI/WT with SCI/CD11b KO mice showed significantly lower expression levels in the latter group (Fig. 1E). Other microglia and myeloid cell markers that we tested included *P2ry12*, *Tmem119*, *CD83*, and *Csf1r*. The markers *P2ry12* (Fig. 1F) and *Tmem119* (Fig. 1G), both of which are abundantly expressed in ramified microglia (58, 59), showed a significant decrease after 1d SCI without genotype effects. The gene CD83 plays a critical role in controlling and resolving immune responses, showed significantly lower mRNA levels in injury groups (Fig. 1H), but no genotypic differences were observed. Finally, the gene that encodes microglial receptor *Csf1r* showed neither injury nor genotype effects (Fig. 1I). In addition, both WT and CD11b KO mice showed marked upregulation of *Gfap* at 1 d post-injury, but no genotype effects were observed, suggesting that genetic depletion of CD11b didn’t affect astrocyte function (Fig. 1J). Collectively, acute SCI leads to increased levels of genes that initiate inflammation-resolving processes and drive myeloid cells towards clearance of damaged tissue and debris, which was not affected by CD11b ablation.

### Depletion of CD11b reduces the number of microglia and infiltration of neutrophils in the injured spinal cord

**Figure 2:**
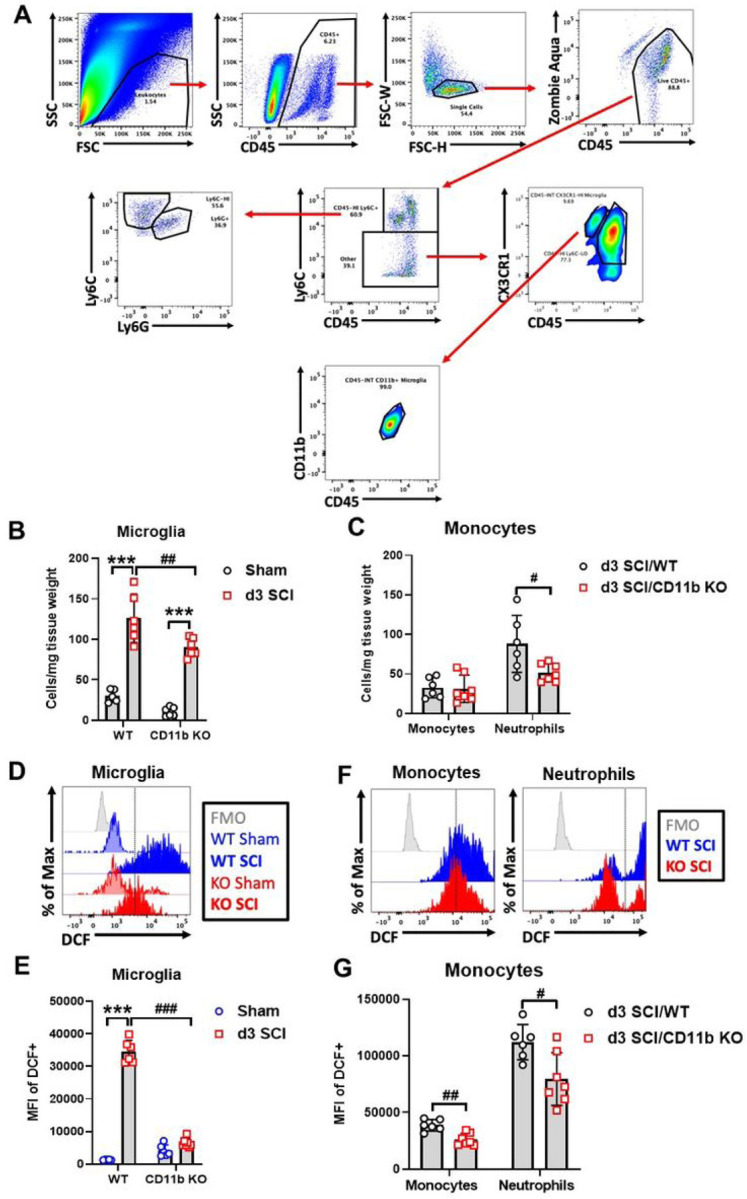
Genetic ablation of CD11b attenuates pro-inflammatory activation of microglia and monocytes at 3 days post-injury. **(A)** Gating strategy for microglia (CD45^int^Ly6C^−^CX3CR1^+^), monocytes (CD45^hi^Ly6C^+^Ly6G^−^), and neutrophils (CD45^hi^Ly6C^+^Ly6G^+^) in CD11b KO mice. **(B-C)** Quantification of microglia (B) and myeloid (C) cell counts are shown. **(D-G)** Reactive oxygen species (ROS) and oxidative stress were measured using DCF. Main effects of genotype and injury were seen in the ROS production levels of microglia (D-E) and myeloid cells (F-G). ***p<0.001 vs. Sham groups. #p<0.05, ##p<0.01, ###p<0.001 vs. SCI/WT. n=5–6 mice/group. Two-way ANOVA followed by Tukey’s post hoc test.

At d3 after SCI when infiltration peaks in the spinal cord, flow cytometry was used to examine the cellular inflammatory response in the lesion site. Due to the absence of CD11b protein in the CD11b KO mice, we used other known microglia and monocyte markers for gating myeloid cell populations. The cell surface marker CX3CR1 is expressed specifically in microglia (60). As indicated in Fig. 2A, microglia were gated on the criteria of CD45 intermediate (int), Ly6C low, and CX3CR1 positive (+), which was validated in WT groups with the traditional gating strategy of CD45^int^CD11b^+^. For infiltrating myeloid cells, the gating strategy of CD45^hi^Ly6C^+^Ly6G^−^ was used to identify monocytes, while Ly6G^+^ indicated neutrophils. Using this new strategy, we were able to observe a significant increase of microglia cell counts at the lesion site of both WT and CD11b KO mice compared to sham groups of the same genotype, however, the number of microglia were markedly lower in SCI/CD11b KO mice (Fig. 2B), suggesting lower levels of injury-induced proliferation. Although the number of infiltrating monocytes remained the same between the two genotypes, CD11b KO mice had significantly lower number of neutrophils (Fig. 2C). Furthermore, ROS production was significantly attenuated in both microglia (Fig. 2D–E) and infiltrating myeloid cells of SCI/CD11b KO (Fig. 2F–G) mice compared to the injured control group, as determined by DCF mean fluorescence intensity. Together, these results demonstrate that CD11b is critical to promoting microglia proliferation, infiltration of peripheral immune cells, and ROS production after SCI.

### CD11b KO mice show robust changes in neuroinflammation-related genes after SCI

**Figure 3:**
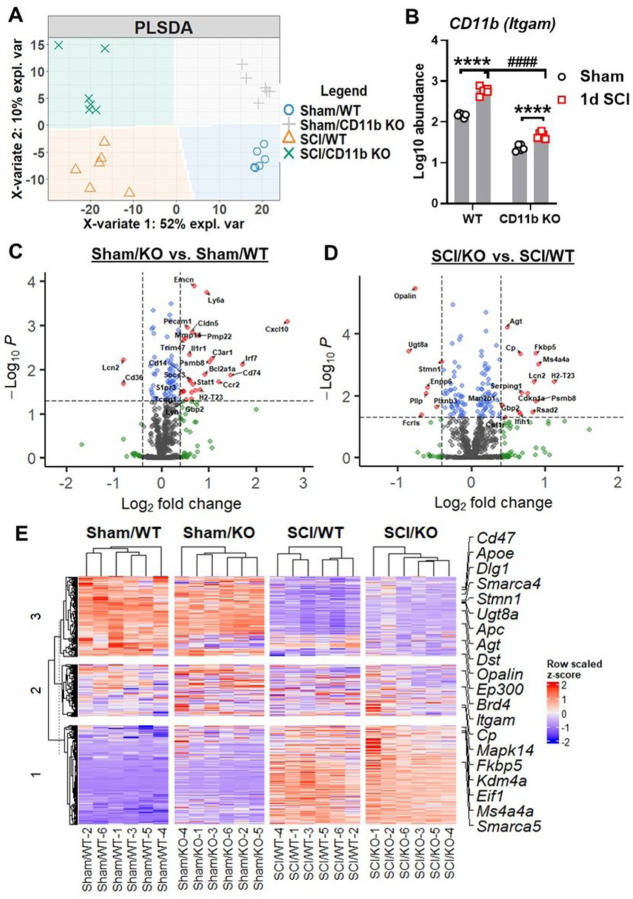
NanoString analysis of spinal cord tissue is performed at 1 day after SCI. **(A)** Partial least square differentiation analysis (PLSDA) was performed with all normalized gene counts from the NanoString neuroinflammation panel. The two main components of variation were captured on the x- and y-axis and showing a clear separation of clusters between the injury and genotype groups. **(B)** Log10 abundance of the *Itgam* (CD11b) gene was obtained from NanoString transcriptional data. ****p<0.0001 vs. Sham groups. ####p<0.0001 vs. SCI/WT. n=6/group. Two-way ANOVA followed by Tukey’s post hoc test. **(C-D)** Volcano plot of differentially expressed genes (DEGs) in the spinal cord of Sham (C) and SCI (D) spinal cord samples with log2(fold-change) larger than 0.4 and log10(P) higher than 1.3 at the cutoff points depicted by dashed lines. **(E)** Heatmap of the top 20 DEGs in pairwise comparison between SCI/*CD11b KO*and SCI/WT. n=6/group.

To determine the consequences of CD11b deletion on acute inflammatory response following SCI, we evaluated spinal cord tissue at the injury site with NanoString Neuroinflammation panel. Partial least square discrimination analysis (PLSDA) was used on all normalized transcription count data to reveal a distinct separation between samples of each group, which was clustered into four quadrants (Fig. 3A). The two main variants of the PLSDA model separated samples by injury (variate 1) and genotype (variate 2), accounting for 52% and 10% of the total variation between samples respectively. Furthermore, the gene *Itgam* regulating expression of CD11b showed significant reduction in the spinal cord of KO mice compared to their WT littermates, which further confirms the validity of the global KO model (Fig. 3B). Pairwise comparison between groups yielded a total of 95 (20 downregulated, 75 upregulated) in Sham/CD11b KO vs. Sham/WT and 123 (54 downregulated, 69 upregulated) genes in SCI/CD11b KO vs. SCI/WT, which indicated robust genotype effects in both baseline and after SCI conditions (Fig. 3C–D). Interestingly, more than double the number of genes showed genotype-induced downregulation in SCI mice than Sham groups. In terms of injury effects, we were able to observe a total of 390 (175 downregulated, 215 upregulated) when comparing 1d SCI vs. Sham in WT (**Fig. S1A**), as well as 385 (184 downregulated, 201 upregulated) genes in SCI/CD11b KO vs. Sham/CD11b KO groups (**Fig. S1B**). As expected, the majority of genes related to neuroinflammation showed dramatic increase at 1d post-injury. Next, we sought to find differentially expressed genes (DEGs) between groups, which yielded the top 20 DEGs in the SCI/CD11b KO vs. SCI/WT comparison set ranked by p-value (Fig. 3E). Amongst the top DEGs with the lowest p-value, *Itgam* and *Mapk14* are involved in the regulation of innate immune response, both of which showed significant reduction in CD11b KO mice after SCI. For cellular function, genes involved with microglia (*Stmn1*, *Dst*) are also downregulated in the injured spinal cord of CD11b KO mice, while the gene *Dlg1* (neurons and neurotransmission) and *Opalin* (oligodendrocyte function) followed similar trends. In addition, four genes (*Apc*, *Agt*, *Fkbp5*, and *Cp*) are involved with the regulation of astrocyte function. Moreover, the genes *Apoe* and *Ep300* are regulators of lipid metabolism, which showed significant upregulation in the spinal cord of CD11b KO mice. There are also several genes that are part of the epigenetic regulation process: *Smarca5*, *Kdm4a*, *Eif1*, *Brd4*, and *Smarca4*. Finally, the genes *Cd47* and *Ms4a4a*, both of which showed significant upregulation in the KO mice, are enriched on the surface of neurons and microglia, respectively.

To confirm the changes observed in NanoString, we performed validation on several DEGs with qPCR. We first examined the mRNA expression levels of four genes that had significant downregulation in NanoString analysis. *Opalin*, a marker for oligodendrocytes (61, 62), showed significant injury-induced downregulation in WT mice (**Fig. S2A**) and even lower levels in CD11b KO mice after SCI. The gene *Fcrls* that modulates Fc receptor-like protein 2, only showed a significant injury-induced upregulation in WT mice (**Fig. S2B**) but not in CD11b KO mice, which was markedly lower at the same timepoint after SCI. The genes *Pllp* and *Ennp6* showed significant downregulation after SCI in both WT and CD11b KO, with no significant differentiation between the two genotypes after injury (**Fig. S2C-D**). We next examined three DEGs that were upregulated in NanoString analysis, the first was *Plekhp1* which showed significant upregulations after injury but no differences between genotypes (**Fig. S2E**). In the case of the genes *H2-T23* and *Fkbp5*, SCI/CD11b KO mice showed significant increases at 1 d SCI compared to sham groups (**Fig. S2F-G**), which was also significantly higher than the SCI/WT group.

**Figure 4:**
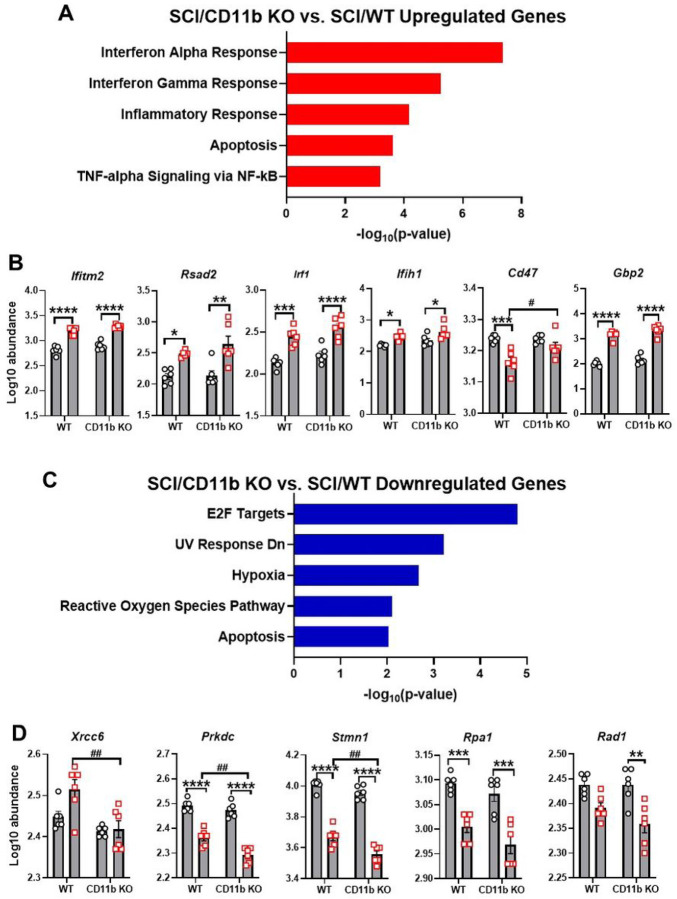
Enrichment analysis reveals potential pathways involved with CD11b-mediated neuroinflammation. **(A-B)** Pathway enrichment analysis of upregulated DEGs (A) with MSigDB Hallmark 2020 and genes involved with Interferon Alpha Response (B). **(C-D)** Pathway enrichment analysis of downregulated DEGs (A) and genes involved with E2F Targets (D). n=6 mice/group. *p<0.05, **p<0.01, ***p<0.001, ****p<0.0001 vs. Sham groups. #p<0.05, ##p<0.01, vs. SCI/WT. n=6 mice/group, Two-way ANOVA followed by Tukey’s post hoc test.

NanoString pathway enrichment analysis of DEGs showed Interferon Alpha Response as the top pathway upregulated in the SCI/CD11b KO vs. SCI/WT comparison set, with Interferon Gamma Response, Inflammatory Response, Apoptosis and TNF-alpha signaling via NF-kB as part of the top enriched pathways (Fig. 4A). Within the top enriched pathway of Interferon Alpha Response, genes included *Ifitm2*, *Rsad2*, *Irf1*, *Ifih1*, *Cd47*, and *Gbp2* (Fig. 4B). Pathway enrichment analysis of downregulated DEGs in SCI/CD11b KO vs. SCI/WT comparison yielded E2F Targets as the top pathway (Fig. 4C). Other downregulated pathways include UV Response Dn, Hypoxia, Reactive Oxygen Species Pathway and Apoptosis. Specific DEGs within the top-downregulated pathway include *Xrcc6*, *Prkdc*, *Stmn1*, *Rpa1*, and *Rad1* (Fig. 4D). Due to the critical role that CD11b plays in the acute immune response to SCI, it is reasonable to assume that the pathways activated by injury in WT mice may be different than those activated CD11b KO mice. Based on this hypothesis, we next examined the injury induced DEGs in WT and CD11b KO groups through pathway enrichment analysis. For WT mice, SCI led to the upregulation of genes in the pathways of Interferon Gamma Response, Inflammatory Response, IL-6/JAK/STAT3 Signaling, TNF-alpha signaling via NF-kB and Apoptosis (**Fig. S1C**). Moreover, SCI also led to downregulation of Reactive Oxygen Species Pathway, E2F Targets, Apoptosis, TGF-beta Signaling, and UV Response Dn in WT mice (**Fig. S1D**). On the other hand, in CD11b KO mice, the top upregulated pathway is IL-6/JAK/STAT3 Signaling, while the top downregulated pathway is UV Response Dn (**Fig. S1E-F**).

**Figure 5:**
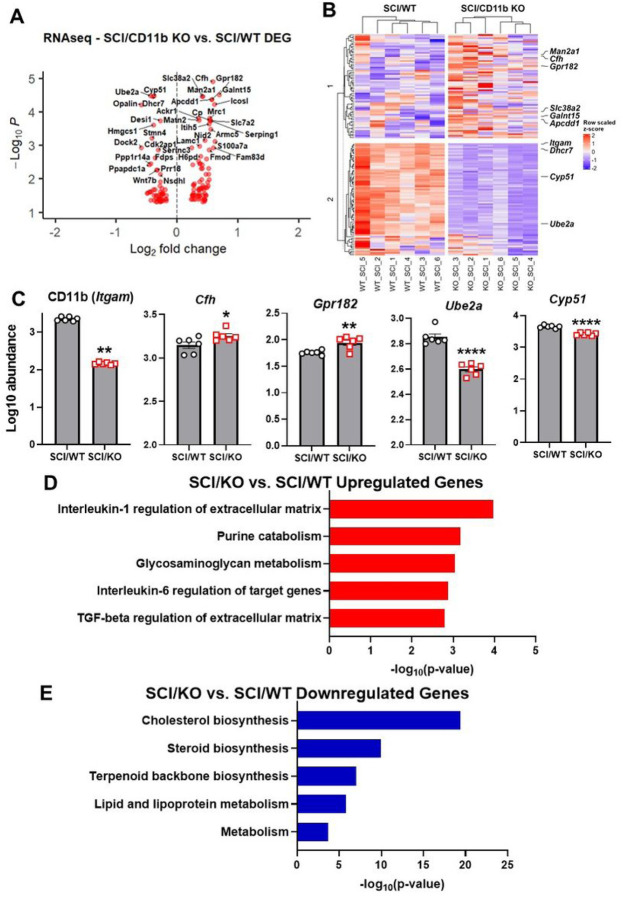
RNA sequencing of injured spinal cord samples at 1d SCI. **(A)** Volcano plot of DEGs obtained from bulk RNAseq. **(B)** Heatmap of top 10 DEGs in SCI/CD11b KO vs. SCI/WT. **(C)** Log10 abundance of top 5 DEGs obtained from pairwise comparison. **(D-E)** Pathway enrichment analysis of up- and downregulated genes. *p<0.05, **p<0.01, ****p<0.0001 vs. SCI/WT. n=6 mice/group, Two-way ANOVA followed by Tukey’s post hoc test.

To further investigate the effects of CD11b ablation on the transcriptomic profile of injured spinal cords, we used bulk RNA sequencing to examine spinal cord tissue at 1d post-injury. Using a cutoff point of FDR < 0.05, we were able to screen a total of 137 DEGs (59 downregulated and 78 upregulated) from SCI/CD11b KO vs. SCI/WT and depict them in a volcano plot (Fig. 5A). In addition, our analysis also yielded the top 10 DEGs (Fig. 5B), with *CD11b, Cfh, Gpr182, Ube2a*, and *Cyp51* being the top 5 genes with the lowest FDR value (Fig. 5C). Moreover, pathway enrichment analysis with the Bioplanet 2019 database showed “Interleukin-1 regulation of extracellular matrix” as the top upregulated pathway (Fig. 5D), while “Cholesterol biosynthesis” is the top downregulated one in the KO mice (Fig. 5E).

In summary, our NanoString and bulk RNAseq results showed upregulation of proinflammatory genes involved with interferon alpha, interferon gamma and other pathways that participate in the development of innate and adaptive immune responses, suggesting that CD11b KO mice had upregulated acute neuroinflammation in response to spinal cord injury, which is a necessary mechanism of defense for the CNS protection.

### Ablation of CD11b improves functional recovery and reduces tissue damage after SCI

**Figure 6:**
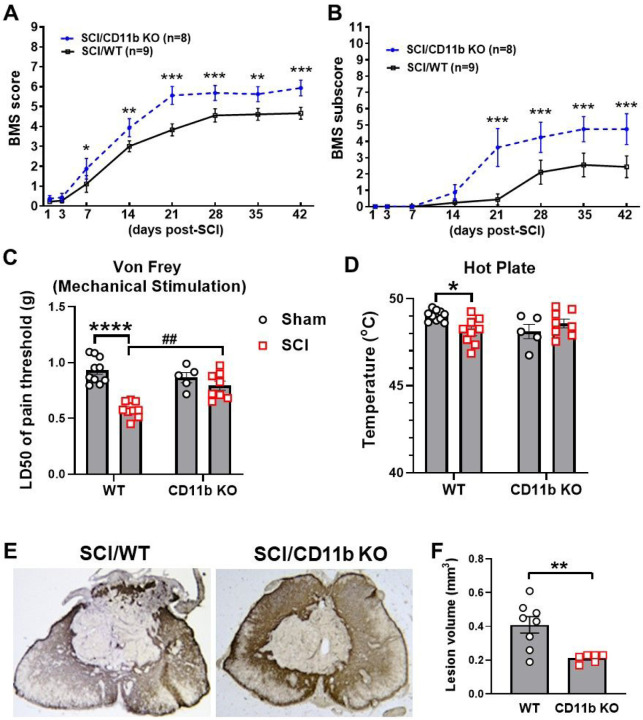
CD11b ablation improves motor and sensory functional recovery following SCI. **(A-B)** BMS scores and subscores were recorded weekly to quantify hindlimb locomotor recovery after SCI. *p<0.05, **p<0.01, ***p<0.001 vs. SCI/WT. n=8–9 mice/group. Repeated two-way ANOVA followed by Holm-Sidak post-hoc analysis. **(C-D)** von Frey monofilament stimulation and hot plate test was utilized for assessment of mechanical and thermal allodynia. *p<0.05, ****p<0.0001 vs. Sham/WT. ##p<0.01 vs. SCI/WT. n=5 for Sham/CD11b KO, n=8–10 for other groups. Two-way ANOVA followed by Tukey’s post hoc test. **(F-F)** Representative images and quantification of lesion volume (LV) at 7 w SCI via GFAP-DAB staining. **p<0.01 vs. SCI/WT. n=6–8 mice/group. Unpaired t-test.

Finally, we assessed behavior and histopathology to determine the chronic impact of CD11b ablation on recovery. Weekly assessment of BMS scores and subscores showed that SCI/CD11b KO mice were recovering at a faster rate than their WT littermates (Fig. 6A–B). Starting from as early as 7 days post-injury, the average score for the SCI/WT mice was 1.11 ± 0.423, indicating that most WT mice within the group only showed slight ankle movement. At the same time point, SCI/CD11b KO mice had an average score of 1.875 ± 0.524, which is indicative of extensive ankle movement but no plantar placement. This dramatic genotype difference at 7 d post-injury (n = 9 for WT, n = 8 for CD11b KO, p = 0.025) was the start of a persistent trend that would continue for 6 weeks (p < 0.001), after which both injury groups appeared to have reached a plateau for motor function recovery. The average BMS scores at the plateau period was 4.6 for WT mice, indicating that mice had occasional or frequent plantar stepping. In contrast, CD11b KO mice had an average BMS of 5.7 to 5.9, which suggests that animals had frequent or consistent plantar stepping. Moreover, BMS subscores also reflected consistent plantar stepping and better coordination. We detected a significant main effect of genotypes [F(1, 15) = 5.549, p = 0.033 for BMS scores and F(1, 15) = 5.525, p = 0.033 for BMS subscores].

Next, we tested whether CD11b is involved in the development of post-injury allodynia evoked by mechanical and thermal stimuli. At 6 w following SCI, mice that regained adequate locomotor function to be able to withdraw a hind paw from a stimulus were selected for further nocifensive behavioral testing. In both tests of nociceptive behavior, there was no difference in mechanical/thermal threshold between the sham groups of either genotype (Fig. 6C–D). After SCI, the 50% mechanical pain threshold of SCI/WT was considerably lower than their uninjured counterparts (Fig. 6C), whereas CD11b KO mice showed little difference between sham and injury groups. Further examination of results demonstrated that the 50% pain threshold of SCI/CD11b KO mice was significantly higher than SCI/WT mice at 6 w post-injury (Fig. 6C), which indicates that hyperesthesia was effectively alleviated by CD11b genetic ablation. In hot plate test, the temperature threshold for WT mice was significantly decreased after SCI (p < 0.05 vs. SCI/WT, Fig. 6D), but no differences were observed between the sham and SCI groups of CD11b KO mice, which further confirms the attenuation of SCI-induced allodynia. After completion of the behavioral tests, we examined tissue damage by lesion volume (LV). Quantification of lesion volume by unbiased stereology showed a much smaller area of glial scarring in CD11b KO compared to their WT littermates (p < 0.01 vs. SCI/WT, Fig. 6E–F). Taken together, the results indicate that CD11b depletion improves recovery after SCI, which is associated with reduced tissue damage.

**Figure 7:**
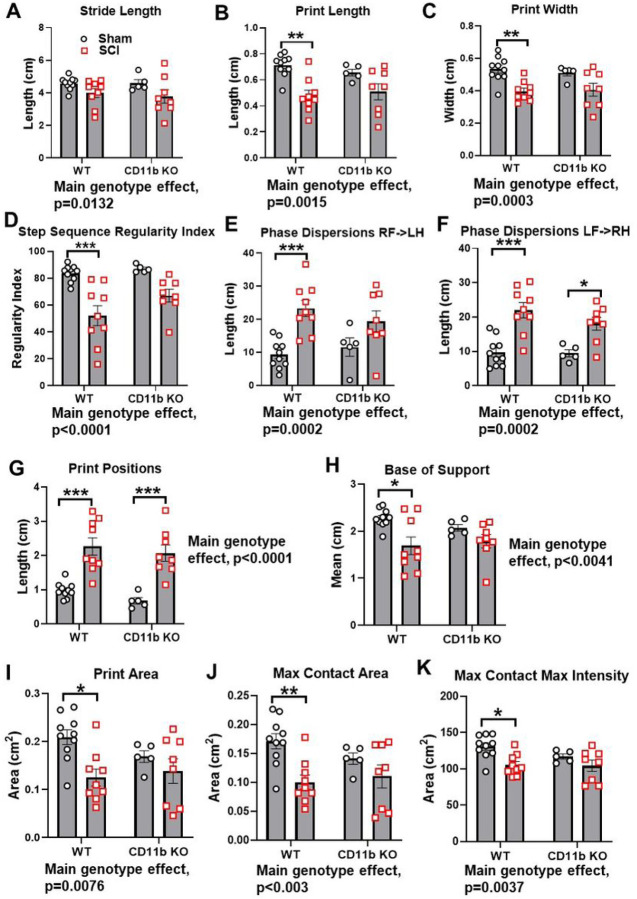
CD11b KO mice showed less deficits of motor coordination after chronic SCI. Catwalk automated gait analysis was used to assess stride length **(A)**, print length **(B)**, print width **(C)**, step sequence regularity **(D)**, phase dispersions **(E-F)**, print positions **(G)**, base of support **(H)**, print area **(I)**, max contact area (J), and max contact max intensity **(K)**. stance patterns. *p<0.05, **p<0.01, ***p<0.001 vs. Sham groups. n=5 for Sham/CD11b KO, n=8–10 for other groups. Two-way ANOVA followed by Tukey’s post hoc test.

For further assessment of motor coordination, we used Catwalk XT gait analysis to examine fine motor differences beyond that recognizable by BMS scores. Stride length is the distance between successive placements of the same paw (Fig. 7A), which showed significant main genotype effect between groups [F (1, 28) = 6.130, P = 0.0196], but no injury effect. Measurement of print length and width also found significant main genotype effects [F (1, 28) = 18.03, P = 0.0002 for print length, F (1, 28) = 17.29, P = 0.0003 for print width], along with marked decrease of both parameters in WT mice following SCI (p < 0.01, Fig. 7B–C), Representative foot print images are indicated in **Fig S3**. Next, we evaluated motor coordination with regularity index, a parameter that tracks the order of paw placement in a step cycle (Fig. 7D). A single step cycle is defined as each of the four paws being placed on the walking surface in sequence, which was analyzed by attributing each set of steps into either a normal stepping pattern or abnormal gain. The result being a percentage of normal stepping out of all step cycles analyzed. As expected, the step sequence regularity index was significantly lower in SCI/WT mice compared to Sham/WT (p < 0.01 vs. Sham/WT, Fig. 7D, S4), indicating clear deficits in motor coordination, but this decrease was not significant in CD11b KO mice. Phase dispersions, a parameter that describes the temporal relationship between placement of two paws within a step cycle, was used to measure inter-paw coordination (Fig. 7E–F). Assessment of the phase dispersions between right forepaw (RF) and left hind paw (LH) yielded significant increase in WT mice following SCI (p < 0.001, Fig. 7E), but the deficits in CD11b KO weren’t significant. On the other side, which examines the diagonal dispersion of left forepaws (LF) and right hindpaws (RH), deficits from SCI could be observed in both WT (p < 0.001) and CD11b KO mice (p < 0.05, Fig. 7F). Moreover, bother parameters showed significant main genotype effects [F (1, 28) = 18.58, P = 0.0002 for RF->LH; F (1, 28) = 31.56, P < 0.0001 for LF->RH]. Print position is defined as the distance between a pair of hind paw and forepaw of the same side. Ideally, healthy C57BL/6 mice should be able to place their hind paw next to the location of the forepaw that has just been lifted from the walkway. Following SCI, the print positions of both WT and CD11b KO mice were significantly increased (p < 0.001, Fig. 7G), while also showing a genotype main effect [F (1, 28) = 44.44, P < 0.0001]. However, pairwise comparison of SCI/WT and SCI/CD11b KO yielded no statistical significance. Hindlimb base-of-support is a parameter that measures the average width of the track (distance between RH and LH) made by the animal, in which the farther apart the feet are placed during locomotion, the less likely the animal is to fall and the larger the base-of-support (BOS). Following SCI, WT mice showed significant decrease in hindlimb BOS (p < 0.05, Fig. 7H), indicating a lack of coordination and trunk stability. We next examined print area, max contact area and max contact max intensity, which could reflect spontaneous pain activity in mice (Fig. 7I–J). The SCI/WT group showed a significant decrease in all three parameters, which suggests reduced contact with the Catwalk surface and the potential presence of spontaneous pain.

## Discussion

In the present study, we examined the role that the major integrin CD11b/CD18 (Mac-1, CR3, αMβ2) plays in the acute inflammatory response after SCI, along with its contribution to locomotor dysfunction and neuropathic pain. Our findings show that experimental SCI causes pro-inflammatory activation of microglia/macrophage, characterized by qPCR, flow cytometry, NanoString, and bulk RNAseq analysis. Genetic deletion of CD11b gene further increases the neuroinflammation in the injured spinal cord tissue. Importantly, CD11b KO mice show significantly improved locomotor functional recovery and reduced cutaneous hypersensitivity to mechanical and thermal stimuli, which were associated with reduced tissue damage. Thus, our data suggest that the CD11b gene may play a deleterious role in the recovery of neurological functions following SCI by suppressing the beneficial acute inflammation.

Using qPCR, we first observed a significant increase of CD11b mRNA in the acute and sub-acute phase of SCI. This is consistent with studies that examined the temporal activation of microglia and macrophages after SCI (18, 63). Using CD11b KO mice, we observed higher expression levels of genes involved in the activation of myeloid cells and their associated inflammatory responses. One of the genes that we examined, *Cxcl10*, regulates a chemokine that actively participates in the initiation of microglial activation following injury (57). Studies have shown that microglial activation in the early stages of SCI has beneficial effects in debris clearance, working with reactive astrocytes to form a glial scar at the lesion site and promoting neuronal regrowth (64–66). We further observed lower expression levels of *Trem2* and *Gdf15* mRNA in the lesion area of CD11b KO mice. High levels of *Trem2* have been reported to be a sign of poor prognosis in Alzheimer’s disease (AD) and a mediator of microglia hyperactivation in neuropathic pain (67–69). Whereas *Gdf15*, a mediator of macrophage inhibitory cytokine-1 (MIC-1), has been reported to an important marker of mortality in the aged population and stroke patients (70–72). Although reports have also identified myeloid *Gdf15* as a potential mediator for regenerative inflammation (73, 74), the acute timepoint of our data excludes regeneration from the scope of study. Taken together, these results suggest that microglia/macrophage in CD11b deficient mice demonstrated an inflammation-resolving phenotype that facilitated debris clearance.

The surface adhesion receptor CD11b is routinely used in flow cytometry and other techniques as a well-known marker of the microglia/macrophage population. In the current study, the genetic ablation of CD11b/CD18 has created a unique challenge for characterizing the neuroimmune profile of the injured spinal cord. Using a combination of microglia and monocyte markers, we were able to devise a novel gating strategy for identifying the cell populations. In this gating strategy, antibodies against CX3CR1 were used to differentiate resident microglia from infiltrating monocytes. Furthermore, differentiation of monocytes and neutrophils was achieved using Ly6C, a surface marker expressed in most blood-borne cells of monocytic origin (75). Using Ly6C and Ly6G, Saiwai et al was able to identify a population of infiltrating monocytes that promotion inflammatory resolution after SCI (76). Similarly, we used Ly6C and Ly6G to separate monocytes from neutrophils. Following initial gating of Ly6C, we used Cx3CR1 to isolate resident microglia from macrophages. Early studies that utilized in situ hybridization demonstrated that the majority of Cx3CR1 + cells in the spinal cord of experimental autoimmune encephalomyelitis (EAE) rats were microglia (60). The high specificity of Cx3CR1 to microglia is further confirmed by later studies that developed Cx3CR1-cre lines as a tool for genetic manipulation of microglia (77, 78).

By using flow cytometry to examine the injured spinal cords’ inflammatory profile, our data also demonstrated that CD11b KO mice had less microglia accumulation and reduced infiltration of macrophages and neutrophils from the blood circulation following SCI, along with lower ROS production. To this end, we first showed that SCI/CD11b KO had significantly lower expression levels of *Trem2* than their WT counterparts. The *Trem2* gene modulates the expression of triggering receptor expressed on myeloid cells-2 (Trem2), which has been shown to be upregulated in the serum of SCI patients (79), control microglia hyperactivation in a model of progranulin deficiency (69), along with the transition of microglia towards a proinflammatory phenotype and exacerbation of neuropathic pain (67). Moreover, a study comparing the mechanisms of inflammatory responses between traumatic and neurodegeneration injuries found that Trem2 plays a major role in the determination of phenotype (80). In our qPCR results, we also observed downregulation of the oligodendrocyte marker *Opalin*, which is required for promotion of oligodendrocyte growth and differentiation (61, 62). One possible explanation for decreased *Opalin* levels is enhanced phagocytosis and cleanup of damaged cells at the acute stage of SCI.

Consistent with the results from flow cytometry, both NanoString and RNAseq showed that CD11b KO mice exhibited significant upregulation of pro-inflammatory transcriptomic factors and genes involved in phagocytosis. Pathway enrichment analysis revealed interferon alpha as the top pathway involved in upregulated DEGs for SCI/WT vs. SCI/CD11b KO. A recent study on chronic constriction injury in rats demonstrated that interferon alpha can improve their mechanical pain threshold, possibly having an analgesic effect (81). Amongst the genes upregulated in SCI/*CD11b KO* mice compared to their WT littermates, Cd47 has been to be instrumental to the phagocytosis engulfment of apoptotic neurons, as CD47 KO mice exhibit excessive synaptic pruning (82). Moreover, the downregulation of E2F targets coincides with our lab’s prior findings on cell cycle-related genes and neuroinflammation after SCI (83). Combined with the attenuation of allodynia observed in the present study, these results provide a possible mechanism for neuroprotection in CD11b KO mice. Thus far, we are the first to study the consequences of CD11b genetic ablation in a rodent model of traumatic SCI. In other models of neuronal injury, studies have shown that CD11b is critical in the development of experimental autoimmune encephalomyelitis and myelin phagocytosis (84, 85). In neutrophils, studies show that CD11b regulates adhesion and recruitment in pathologic inflammation (86, 87).

Despite advancements in emergency medicine and higher survival rates, no effective therapy has emerged for SCI patients, who often suffer from temporary or permanent motor and sensory deficits. Immunotherapy strategies have achieved exciting progress in treating many CNS degenerative disorders such as Alzheimer’s disease (AD) (88–90), Parkinson’s disease (PD) (91–93), multiple sclerosis (MS) (94–96) and have even been adapted for traumatic brain injury (97). Our study shows for the first time that genetic ablation of CD11b could suppress oxygen free radical-associated tissue injury by microglia/macrophages/neutrophils while promoting neuroprotection. A study by Wolf et al. showed that ligand-specific blockade of CD11b with CD40L could target regional inflammation without affecting host defense (98). Thus, one possible future direction would be the intrathecal injection of ligand-specific antagonists, which has more therapeutic and translational value. Based on the positive results shown in this study, delayed administration of CD11b antagonists may attenuate allodynia following SCI.

## Conclusion

Taken together, we showed that CD11b KO significantly altered the ability of microglia/monocytes to migrate and infiltrate into the lesion site following SCI. We further demonstrated that SCI/CD11b KO mice had a pro-inflammatory profile and enhanced phagocytic activity with lower ROS production, thus promoting functional recovery following SCI. This was further supported by results from the NanoString Neuroinflammation panel and bulk RNA-seq. In chronic SCI, behavior assessment showed reduced motor deficits in CD11b KO mice compared to their WT littermates, along with mechanical and thermal allodynia attenuation. These findings suggest that CD11b is a major pathophysiological factor in SCI-mediated inflammatory response and related neurological impairments.

## Data Availability

All data needed to evaluate the conclusions in the paper are present in the paper and/or the Supplementary Materials.
